# Impact of COVID-19 and Consortium Factors on Mental Health: Role of Emotional Labor Strategies in Achieving Sustainable Development Goals

**DOI:** 10.3389/fpsyg.2022.795677

**Published:** 2022-03-03

**Authors:** Saqib Rehman, Muhammad Ali Hamza, Adeel Nasir, Aman Ullah, Nabeela Arshad

**Affiliations:** ^1^Department of Management Sciences, Lahore College for Women University, Lahore, Pakistan; ^2^UVAS Business School, University of Veterinary and Animal Sciences, Lahore, Pakistan; ^3^Faculty of Business and Law, CQ University, Melbourne, VIC, Australia; ^4^Hailey College of Banking and Finance, University of the Punjab, Lahore, Pakistan

**Keywords:** COVID-19, SDG 8, mental health, fear of economic crisis, perceived job insecurity, surface acting, deep acting

## Abstract

The COVID-19 pandemic that began in 2019 has created an acute fear of economic crisis, and people have experienced the state of perceived job insecurity. Several measures were taken to control this deadly pandemic, but it still affected the majority of global operational activities. This study addresses the United Nation’s Sustainable Development Goal (SDG) number 8 that relates to decent work and economic growth. This quantitative study examines the impact of fear associated with economic crisis and perceived job insecurity on mental health with the moderating effect of surface and deep acting. Surface acting is displaying fake emotions, and deep acting is modifying inner feelings according to the required emotions. This study used sample data from private-sector employees and applied SmartPLS for structural model assessment. As many organizations took more challenging decisions to sustain their business operations, the study therefore analyzes the impact of the pandemic on private sector employees. The two main findings of the study are: (i) surface acting moderates the relationships of fear of economic crisis and perceived job insecurity with mental health and declines the impact of both on mental health, (ii) while deep acting negatively moderates the relationships of fear of economic crisis and perceived job insecurity with mental health and improved mental health even in the presence of both. The study highlighted the importance of deep acting at workplaces to sustain employees’ mental and psychological stability. Organizations could introduce emotional labor strategies and strengthen the mental health of their employees against the underlying fear of economic crisis and perceived job insecurity.

## Introduction

At the end of 2019, an unknown pneumonia was detected in Wuhan, Hubei province of China, that was caused by a microbial pathogen. Subsequently, a new virus was identified and named coronavirus or COVID-19. Globally, this infectious virus has become a threat to the general public and consequently other countries cut themselves off from China. After that, on January 30, 2020, the World Health Organization (WHO) declared the occurrence of the new coronavirus as an epidemic and imposed a public health emergency of international concern (Gao and Liu, 2020). The new coronavirus spread in a drastic and unpredictable manner not seen before in recent history. Serrano-Ripoll et al. (2020) highlighted the impact of epidemic outbreaks on mental and psychological health through the consortium effect of factors like lack of social support, stigmatization, working in a high-risk environment, and specific occupational roles. In addition, Nicola et al. (2020) stated that due to the new coronavirus, billions of people were facing financial crisis in the form of loss of income and sustained social isolation in the form of full or partial lockdowns, which affected their mental health.

To respond to such a huge challenge, governments in each country organized awareness programs, i.e., public service messages about precautionary measures to spread information about the possible effects of such a contagious pandemics on a large scale (Lu et al., 2020). Different studies have demonstrated that people who directly suffered from this infectious virus retained a higher rate of depression, anxiety, and other psychological problems (James et al., 2020; Wilson et al., 2020). These psychological problems are caused by fear of potential exposure to infection on one hand and fear of financial instability on the other. In both cases, the mental health of people is at danger and authorities seem to be compromising on or ignoring these facts. Perceived job insecurity and fear of economic crisis are significant psychological reasons for the increased mental anguish from which the majority of the world’s population experienced during the pandemic (Gardner and Moallef, 2015; James et al., 2020; Serrano-Ripoll et al., 2020). In April 2020, an increase of 14.7% in the unemployment rate was observed, which is higher than ever before in history (Xue and Li, 2020). Due to false perceptions and acceptance of unknown fears, people either lost their jobs or job compensations like benefits, promotions, and incremental advantages (Margerison-Zilko et al., 2016; Forbes and Krueger, 2019).

During this stressful period, people took additional jobs to meet their household expenses (Forbes and Krueger, 2019). Such circumstances caused anxiety and depression. Over time, COVID-19 has contributed to mental and psychological instability and society’s distress. Earlier research proved that pandemics directed the general public toward experiencing stress disorders closely related to depression and anxiety (Liang et al., 2020). More often, globally, it was acknowledged that top-level organizational managers must understand their employee’s mental well-being status. As employees’ mental and psychological stability fluctuates under unusual conditions, the management of these circumstances is an essential ingredient of organizational performance. Therefore, managers have to work diligently and devise strategies to manage emotional labor and prevent employees from being mentally exhausted.

The United Nations outlined 17 SDGs to provide decent healthy lives for everyone on the planet by 2030. The major challenge is to achieve these development goals in their entirety. The challenges are harder for developing economies (Population Matters, 2021). SDG 8 deals with decent work and economic growth; the goal’s scope is so broad that it deals with the economic growth potential of developing economies. SDG 8 requires an improved, healthy, and innovative working environment that contributes significantly to economic growth. It entails workforces’ demographics, which provide growth potential for the economy. Mental health is the critical determinant of workforce well-being. COVID-19 with the additional effect of fear of economic crisis and perceived job insecurity has affected people’s mental health. However, emotional labor strategies provide implications for the workforces’ mental health, leading to the impediment of achieving the decent work objective of SDG 8. Emotional labor strategies are emotions that employees can display at work to affect emotions. Both emotional labor strategies require you to exhibit a smile on your face or display friendly behavior to retain customers. Both can be improved by training the employees (Rehman et al., 2021). Therefore, both can significantly impact mental health due to their moderating nature. Deep acting is the display of desired emotions by changing inner emotions, and it includes feelings and expressions, reducing the negative impact on mental health. Comparatively, employees modify their facial expressions in surface acting without changing their inner feelings. It is about faking positive emotions and suppressing negative emotions (Chi and Grandey, 2019). Although it seems complicated to achieve the world development goals by 2030 during a pandemic, this study provides ways to curb these impediments. The study aims to devise a model to test the impact of fear of economic crisis and perceived job insecurity on mental health with the moderating effect of emotional labor strategies.

This pandemic is unique in nature. However, people have to account for depression and anxiety to tackle such circumstances, as the cure for this contagious disease is still in progress by health practitioners. Evidence proved that pandemics affected mental and psychological health unsympathetically, leading people toward stress, anxiety, and depression as each organization seemed to be victimized by the influential impact of COVID-19 (Khanchel, 2021). In this regard, organizations took corrective measures to sustain their business operations and switched to cost-cutting formats like reduction in administrative expenses, cutting off existing employees’ pay, and termination of employees from both temporary and permanent positions. Prior studies showed that such circumstances affect the performance and mental stability of employees.

This pandemic caused severe fear of the economic crisis faced globally. Whenever crises are spread, evidence shows that people face reduction in income due to cut-offs in their earnings, and with the extended existence of pandemics, these reductions led people to different kinds of psychological fears like fear of an economic crisis.

The primary purpose of conducting this quantitative study is to document the theoretical as well as empirical gap between fear of economic crisis and perceived job insecurity on mental health via moderation of emotional labor strategies, i.e., surface acting and deep acting, and the entire conceptual model is done in the context of COVID-19. Deep acting is the display of desired emotions by changing inner emotions, and in surface acting, employees modify their facial expressions without changing their feelings. In the guidance of the research gap, this study attempted to address the following research questions: (i) Does the fear of economic crisis and perceived job insecurity impact employees’ mental health during COVID-19? and (ii) Do emotional labor strategies play a role in mitigating the impact of fear of economic crisis and perceived job insecurity on employees’ mental health? The study’s findings may likely contribute to the development of organizational strategies, programs, and policies, and promote equitable access by helping managers prevent their employees from worrying about perceived job insecurity and fear of economic crisis, especially in times of crises or unusual circumstances. Secondly, this study will contribute a comprehensive addition to the body of existing literature as corporate and service sectors are never studied by the researchers from the emotional labor strategies’ viewpoint in the context of COVID-19.

This quantitative study will give ample evidence to account for the significant impacts of a pandemic on corporate and service sectors to analyze the people’s financial, mental, and psychological sufferings under unusual circumstances. The research was carried out in the contextual framework of Pakistan, where the people are in a state of perceived job insecurity and fear of economic crisis due to the global pandemic. As most countries are facing the same crisis, this study will also set useful parameters for other developing countries. The research will help people sustain their current jobs without engaging with stress or any further mental instability. This study is proposed to set a newer perspective on how people’s mental and psychological stability can be sustained under unusual and unpredictable circumstances, which is an essential ingredient for organizations’ best performance.

Studying this model will extend the literature by directly highlighting the components affecting mental and psychological stability under unusual circumstances. Furthermore, this study provides evidence to understand the factors influencing the mental and psychological sufferings of the private sector. There is a need to deploy strategies to better help employees in pandemics to perform better by diminishing the undue fears and false perceptions.

## Literature Review and Hypotheses Development

Perceived job insecurity is an issue that prevails at the societal level and individual level in difficult times. Employees become more sensitive to their jobs if they perceive that organizations are carrying out lay-offs due to the prevailing situation. This phenomenon has been explained in uncertainty management theory as a guiding principle (Lind and Van den Bos, 2002). This theory explains that employees become more vulnerable and sensitive during uncertain times; therefore, organizations need to be more thoughtful and considerate. Aneshensel (1992) explained the reaction of individuals and their linked families when they face stress, and that those periods of stress and anxiety could badly affect their psychological and mental health. Crises affect everyone in society, and people tackle and respond in varied ways, but the organizations handling difficult conditions should be mature and structured in terms of strategies.

### Fear of Economic Crisis and Mental Health

One of the major threats to people’s general health is the fear of economic crisis (Odone et al., 2018). Over the last couple of years, an extreme increase in mental and psychological suffering has been recorded. As the current ongoing pandemic started in 2019, it continues to be inflated in different countries. At time of the commencement of pandemic, past experiences were being followed; later on, experts concluded that the contemporary pandemic is unique in nature and there is no such precedence available in the literature (Spinelli et al., 2020).

Since it is the most severe fear of economic crisis that has been faced globally in recent history, generally, it is assessed that the consequences of fear of economic crisis are different in nature. Whenever crises are spread abruptly, evidence shows that people face reduction in income, cut-offs to their earnings, and have interruptions in their service delivery places (Glonti et al., 2015). Reduction in income leads people to unemployment and different kinds of mental and psychological sufferings through their congested or low-income budgets. Consequently, people start compromising their internal sufferings at the cost of managing their household expenses (Van Bortel et al., 2016).

Youth unemployment has become an emergent challenge (Elder and Rosas, 2015). According to statistics from the World Bank, the unemployment rate of developing countries was 5.373 in 2019, while it became 6.471 at the end of 2020 with an increase of 1.098 (The World Bank, 2021). Research evidence says that employees’ mental health can adversely be affected by the economic crisis prevailing in the country (Frasquilho et al., 2015; Silove et al., 2017; Wang et al., 2021). Many studies also linked income loss, lack of finances, and unemployment with employees’ mental health (Warr, 1987; Taris, 2002; Paul and Moser, 2009). Thus, a hypothesis with a positive relationship has been proposed based on the above arguments.

H1: *There is a positive relationship between fear of economic crisis and mental health.*

### Perceived Job Insecurity and Mental Health

Over the last few years, globalization has put a strain on the labor market. The ongoing pandemic has triggered an abrupt crisis in all industries; these industries include finance and insurance, health care, social assistance, construction, art, entertainment, tourism, industrial production, and manufacturing units (Chang et al., 2020). During the pandemic, such industries were affected entirely or partially by lower demands, lower production, and higher cost of raw materials. As a result, organizations were compelled to take retributive decisions by cutting off their expenses for future standings; such decisions directly concerned the lower and middle-income level employees. Therefore, these decisions resulted in job loss or increased perceived job insecurity among the workers (Griep et al., 2016; Chapman et al., 2020; Blanuša et al., 2021).

Job insecurity is connected with stress and negative emotions (Ashford et al., 1989; Lim, 1996). Therefore, enough evidence is available to demonstrate that a person’s perceived job insecurity is linked to stress and anxiety (Schaufeli, 2016; Menéndez-Espina et al., 2019; Zhang et al., 2020). Furthermore, it has become worse for those who were working with industries those are highly affected by complete or partial shutdowns (Wilson et al., 2020). Thus, a hypothesis with a positive relationship has been proposed based on the above arguments.

H2: *There is a positive relationship between perceived job insecurity and mental health*.

### The Moderating Role of Emotional Labor Strategies

Emotional labor strategy is associated with the control of feelings to generate both facial and bodily displays that can be observed visibly. The conceptual framework of emotional labor has been segregated into three major categories: (i) surface acting, (ii) deep acting, and (iii) genuine acting (Diefendorff et al., 2005). Emotional labor strategies are the variety of employees’ emotions that employees can display at work to change emotions. Both emotional labor strategies require exhibiting a smile on the face or displaying friendly behavior to retain customers. Both can be improved by training the employees (Rehman et al., 2021). Therefore, both can significantly impact in upgrading mental health due to their moderating nature.

Nowadays, employees’ mental health management is a challenge for organizations (Odone et al., 2018). The last 2 years were challenging, as the world was passing through the state of such a pandemic whose outcomes were unique and diverted many times to date. Despite the availability of numerous studies on viral infections, there was a substantial deficiency in classifying the virus at its commencement phase (Glonti et al., 2015). While the consequences of the pandemic affect all sectors, private sectors were most affected in terms of remunerations, incomes, and benefits. Subsequently, employees have undergone a state of perceived job insecurity and got stress, anxiety, and depression (Wilson et al., 2020).

Surface acting describes how an individual can manipulate or fake emotion according to the required standards set by the organization. If the emotions are genuinely displayed by an individual and can be confirmed by the observer, then it can be said that the set standards are being followed in actual letter and spirit, while the surface acting designates the extent to which one should hide emotions while dealing with the customers (Chi and Grandey, 2019; Rehman et al., 2021). Such fake display of emotions does not alter the inner feelings adequately but used to meet the display rules in organization’s context.

However, available literature and studies or theories have no prescribed evidence of genuine acting, the third category of emotional labor strategies (Diefendorff et al., 2005). In our current study, the first two strategies holding theoretical bases are considered for their valuable impact on mental and psychological suffering.

Deep acting designates the extent to which one keeps trying to improve his mood and deals with the inner-self or emotions (Chi and Grandey, 2019). An individual displays their inner feeling purely while dealing with customers (Lee and Chelladurai, 2018; Rehman et al., 2021). It is experiential that individuals’ positive emotions have been overlapping with the negative ones so that they may not be able to approach the standards set by the organization (Grandey, 2000). Thus, based on the above arguments, the following hypotheses are stated:

H3a: *Surface acting moderates the relationship between fear of economic crisis and mental health.*
H3b: *Surface acting moderates the relationship between perceived job insecurity and mental health.*
H4a: *Deep acting moderates the relationship between fear of economic crisis and mental health.*
H4b: *Deep acting moderates the relationship between perceived job insecurity and mental health.*

**FIGURE 1:**
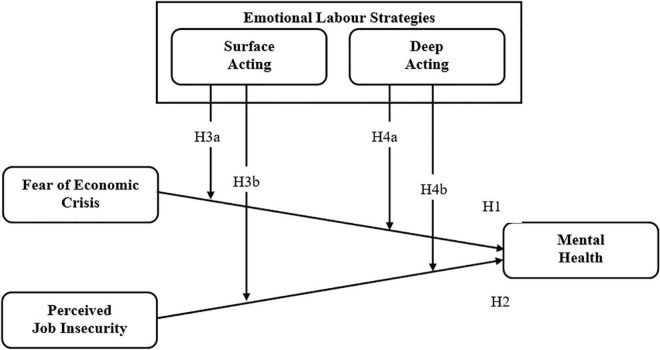
Conceptual model.

In light of the above literature review and development of proposed hypotheses, a conceptual model (Figure 1) has been framed out.

## Materials and Methods

### Participants

Primary data is collected through a questionnaire survey technique and data is collected from classified staff and administrative professionals of the underlying private sector. These professionals are rendering their services in the banking sector, call centers, educational institutions, and the paramedical institution. All the staff and administrative professionals have responded as per their appreciable cooperation through online questionnaires where the condition of anonymity has been assured. The Google questionnaire was also sent directly to potential respondents for participation in the study. Some of the respondents were associated with service call centers, whose jobs are linked with specific weekly, monthly, quarterly, and annual sales-based targets, and decline in their net sales have raised job insecurity. This economic class stratum is more vulnerable to the ongoing pandemic because most potential customers were turned into cautious ones. Generally, the future is still unpredictable under updated circumstances.

### Procedure

To determine the required number of respondents of the data set, two methods were used. Firstly, the response rate is calculated by “G*power” of version (3.1.9.2) with 99% power, Multiple Correlation (R) with 0.30, and at the two-tailed significant levels of 0.05. In this way, the figure of 110 responses was determined. The purpose of using this software is to assure the suitable predictable power of the constructs of the underlying population. Second, the response rate is also calculated by the 10:1 criterion (Hair, 2010). By adopting this strategy, the numbers of items of all the constructs are multiplied by 10. This current study has four variables holding 32 items (32 × 10 = 320), which were acknowledgeable for precise and smooth analysis. The data for this quantitative study is collected through a simple random sampling technique of the underlying population. All the private service sector population had an equal chance to contribute their valuable participation to establish the authenticity of the study. Using simple random sampling technique is cost-effective and accommodates the researcher from all possible time constraints. Four hundred seventy staff members and administrative professionals were supposed to participate. Based on these respondents, 320 participants responded, and the response rate was 68%, whereas the remaining 32% did not contribute to the study.

### Survey Development

Respondents were requested to record their responses to the English language survey questionnaire. The online “Google Form” survey technique was carried out by considering the severe third wave of COVID-19. In the current and ongoing scenarios, where people are getting exposed to this viral infection more rapidly, Google Forms is an excellent and comprehensive tool that enables researchers to access the respondent in a more precise manner.

The questionnaire was prepared through Google Form and circulated online. To make the questionnaire more precise, an introductory paragraph at the commencement of the questionnaire was mentioned to motivate participants and give them assurance regarding the anonymity of provided data. Online modes were taken to circulate the information regarding participation in the study, such as Facebook, Messenger, and WhatsApp groups; upon receipt of consent by probable participants, the questionnaire link was shared. The questionnaire survey was conducted in April 2021.

Furthermore, it was mandatory to answer all the items of contributed latent constructs; by taking this initiative, the prepared database was free from missing values. Data was input to Excel and imported on Statistical Package for Social Sciences (SPSS) of version 24 for screening. Using SPSS, missing values, outliers, skewness and kurtosis, and standard data distribution were tested. After that, the retrieved database was imported into SmartPLS of version (3.2) for measurement and structural model assessments.

The entire questionnaire was segregated into five sections; the first section holds the data related to demographical measures, including gender, age, qualification, and individual job level. Its assessment is carried out on a nominal scale. In the rest of the sections, respondents were asked about the constructs of the study, i.e., fear of economic crisis, perceived job insecurity, emotional labor strategies, and mental health carried out through their respective number of items of 4, 7, 8, and 12. The established 5-point Likert scale was used for four latent constructs to measure their intensity. In the online data collection, respondents were free to rate the extent of their own feelings and emotions. There were no missing values or responses, as the respondent needed to answer all the given questions to proceed further.

Most of the time, collected data from a single source causes common method biases. To precisely control the common method bias, the questionnaire was evaluated by applying the different methods to ensure inconspicuousness and that the answers are in the form of their respective demographical category and respondents are free to choose purely according to their feelings (Podsakoff et al., 2003; Hao and Lirong, 2004).

#### Fear of Economic Crisis

Fear of economic crisis was measured using a four-item scale [by Giorgi et al. (2015)] on a 5-point Likert scale from strongly disagree (1) to strongly agree (5).

#### Perceived Job Insecurity

Perceived job insecurity was measured using a seven-item scale [by Kinnunen et al. (1999)] on 5-point Likert scale from strongly disagree (1) to strongly agree (5).

#### Mental Health

Mental health was measured using a 12-items scale taken from the General Health Questionnaire (GHQ-12), which was designed to diagnose the psychiatric disorder among people [by Montazeri et al. (2003)] on a five-point Likert scale from strongly disagree (1) to strongly agree (5).

#### Surface Acting

Surface acting was measured using a four-item scale [by Diefendorff et al. (2005)] on a five-point Likert scale from strongly disagree (1) to strongly agree (5).

#### Deep Acting

Deep acting was measured using a four-item scale [by Diefendorff et al. (2005)] on a 5- point Likert scale from strongly disagree (1) to strongly agree (5).

### Data Analysis

Approximately 470 people were reached and 320 contributed to the study and filled out the survey. As shown in Table 1, majority of the respondents of the study were male, at 73% (*n* = 320), while females held the percentage of 27 (*n* = 86). According to age, 75% of respondents belong to the age group of (25–30) while a significant portion of respondents held Bachelor’s degrees and provided their services in middle-level jobs (40 and 83%, respectively).

**TABLE 1:** Respondents’ profile.

Demographic characteristics		%
**Gender**		
	Male	72.8
Age	Female	27.2
	25–30	75.3
	31–35	3
	36–40	16.7
	41–45	4
Academic qualification	Above 46	1
	Intermediate	0
	Bachelor’s	50
	Masters	40.1
	MPhil	9.3
Job level	Diploma	0.6
	Low level	8
	Middle level	83.3
	Higher level	8.6

This study was conducted by considering the four measures which made up the model. The fear of economic crisis is an exogenous variable measured by four items (e.g., my organization is solid, although there is a fear of economic crisis). Adjustment of factor analysis, values of factor loadings, Average Variance Extracted, and constructs’ reliability values were obtained by running the PLS algorithm (Hair, 2010). The outcome (Table 2) represents that all the values fall under the recommended criterion of having the Cronbach’s alpha value higher than 0.7 while the consistent reliability is higher than the recommended criterion of greater than 0.7. The value of the average variance extracted also falls under the suggested criterion of higher than 0.5 (Nunnally and Bernstein, 1994).

**TABLE 2:** Validity and reliability for constructs.

Constructs	Items	Loadings	CA	AVE	CR
Fear of economic crisis	I am scared that my organization is affected by the economic crisis	0.886	0.801	0.716	0.883
	I am scared that my organization, because of the economic crisis, is subjected to downsizing	0.849			
	The organizational future is unstable (unknown) because of the economic crisis	0.801			
	My organization is solid, although there is an economic crisis	0.115			
Perceived job insecurity	The thought of getting fired really scares me	0.808	0.817	0.646	0.879
	I am worried about the possibility of being fired.	0.890			
	Working hard would keep me from getting fired	0.456			
	If I get fired, I will not know how to tell people	0.711			
	If I do good work, my job will be safe.	0.674			
	I am so worried that I would do almost anything to keep my job	0.777			
	I am worried about the disgrace of being fired	0.731			
Surface acting	I fake a good mood when interacting with customers	0.840	0.806	0.628	0.871
	I put on a “show” or “performance” when interacting with customers	0.785			
	I pretend to have the emotions I need to display for my job	0.811			
	I show feelings to the customers that are different from what I feel inside	0.730			
Deep acting	I try to actually experience emotions that I must show to customers	0.813	0.880	0.731	0.916
	I make an effort to actually feel emotions that I need to show to the customers	0.870			
	I work at developing feelings inside of me that I need to show to the customers	0.906			
	I work hard to feel emotions that I need to show to the customers	0.828			
Mental health	I am unable to concentrate on my work	0.778	0.945	0.644	0.952
	I lost sleep over worry	0.741			
	I felt I am not playing a useful part in things	0.661			
	I felt incapable of making decisions	0.778			
	I felt constantly under strain and stress	0.828			
	I felt that I could not overcome difficulties	0.856			
	I am unable to enjoy day-to-day activities	0.766			
	I am not able to face problems	0.804			
	I am feeling unhappy and depressed	0.847			
	I have been losing confidence in myself	0.831			
	I have been thinking of myself as worthless	0.775			
	I am feeling reasonably unhappy	0.818			

The second construct is perceived job insecurity, measured by putting its seven items under evaluation (e.g., the thought of getting fired rarely scares me). These items enable the respondent to analyze the possible and upcoming uncertainties regarding their prescribed jobs. These items were assessed by a 5- point Likert scale (1 = strongly disagree; 5 = strongly agree). Respondents were asked to rate the insecurities of their jobs under this recommended criterion. Output table constitutes that loadings are above 0.7, the value of AVE is greater than 0.5, while construct reliability is above 0.7 (Fornell and Larcker, 1981); all these mentioned values fall as per the suggested criteria.

Emotional labor strategies play a moderating role in the study; this strategy comprises of surface acting and deep acting. Surface acting assessed how people modified their moods and feelings within an organization. This modification aims to achieve the actual standards developed by the organization. Deep acting was introduced to assess an individual’s inner feelings and thoughts separate to requirements while performing. Output table constitutes that loadings are above 0.7, the value of AVE is more significant than 0.5, while construct reliability is above 0.7 (Fornell and Larcker, 1981); all these mentioned values fall under the suggested criteria.

Mental health was measured by putting the general health questionnaire comprised of 12 items (e.g., I am unable to concentrate on my work). Assessment of concentration, pressure, depression, anxiety, loss of confidence, loss of sleep, and considering oneself as worthless was taken. Respondents were free to rate their mental and psychological sufferings at their optimal level. Output table constitutes that loadings are above 0.7, the value of AVE is more significant than 0.5, while construct reliability is above 0.7 (Fornell and Larcker, 1981); all these mentioned values fall under the proposed criteria.

## Results

In an adjusted model, discriminant validity is used to differentiate the measures of each construct from each other (Urbach and Ahlemann, 2010). According to Fornell and Larcker (1981), discriminant validity can be examined by comparing the potential overlapping of the constructs possible to assess by cross loading method (Chin and Newsted, 1999). Its implementation can be carried out by comparing indicators’ outer loadings with their associated underlying constructs. According to the outcome (Table 3), the diagonal values are higher than their underlying constructs as per its recommended criterion.

**TABLE 3:** Discriminant validity.

Constructs	DA	EC	MH	PJI	SA
DA	0.855				
EC	0.261	0.718			
MH	0.193	0.420	0.791		
PJI	0.267	0.532	0.550	0.71	
SA	0.368	0.334	0.318	0.369	0.793

According to Kline (2011), the threshold value for Heterotrait-Monotrait (HTMT) should be less than 0.85, and the outcome (Table 4) falls under this recommended criterion. Despite HTMT, a confidence interval is also required to assess where the upper and lower confidence interval should not include the digit of 1 (Henseler et al., 2015), also used to confirm discriminant validity.

**TABLE 4:** Heterotrait-Monotrait (HTMT).

Constructs	DA	EC	MH	PJI	SA
DA					
EC	0.333				
MH	0.192	0.5			
PJI	0.309	0.632	0.585		
SA	0.456	0.438	0.345	0.435	

Table 5 shows structural estimates and hypotheses status by defining Beta values, T statistics and p values where values interlinked with H1 (β = 0415, *p* < 0.01) and H2 (β = 0.352, *p* < 0.001) showed that the first two hypotheses are true as both fear of economic crisis and perceived job insecurity have a significant positive impact on mental health.

**TABLE 5:** Structural estimates.

Hypotheses	Beta	*T* statistics	*P-*values	Decision
H1: Fear of economic crisis->Mental health	0.415	2.495	0.01**	Accepted
H2: Perceived job insecurity->Mental health	0.352	4.606	0.001***	Accepted
H3a: Fear of economic crisis × Surface acting->Mental health	0.043	1.980	0.05*	Accepted
H3b: Perceived job insecurity × Surface acting->Mental health	0.132	3.570	0.05*	Accepted
H4a: Fear of economic crisis × Deep acting->Mental health	(0.213)	2.213	0.001***	Accepted
H4b: Perceived job insecurity × Deep acting->Mental health	(0.391)	3.903	0.01**	Accepted

*p < 0.05; **p < 0.01;***p < 0.001.

Next, hypotheses are developed under consideration of the moderating impact of surface acting and deep acting, respectively. H3a (β = 0.043, *p* < 0.05) showed that surface acting moderates the relation between fear of economic crisis and mental health, and H3b described that surface acting moderates the relation between perceived job insecurity and mental health (β = 0.0132, *p* < 0.05), so the proposed hypotheses are accepted. Results showed that surface acting reduced the effect of economic crisis and perceived job insecurity on mental health. As a result, a considerable fall in mental health values seem to be noted. To examine the moderating role of deep acting on the economic crisis and perceived job insecurity to mental health, H4a and H4b were designed. Results showed (β = −0.213, *p* < 0.001) that deep acting negatively moderates the relationship of economic crisis and mental health, and deep acting strategy converted the negative feeling into positive feelings. At the same time, H4b also displayed the same results and described that (β = −0.391, *p* < 0.01) deep acting negatively moderates the relationship of perceived job insecurity and mental health. A positive increase in mental health has been observed by moderating emotional labor strategies.

**FIGURE 2:**
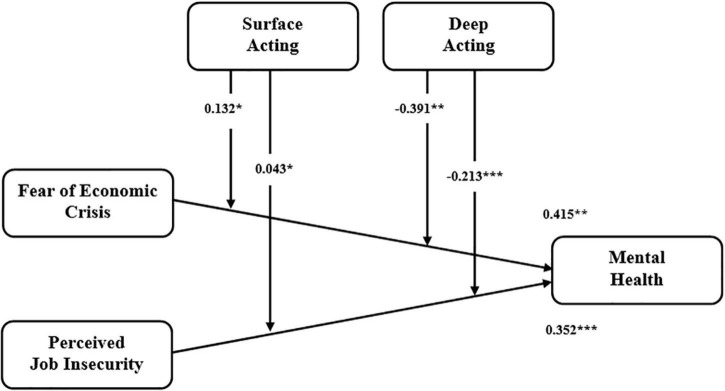
Structural model with path estimates. *p < 0.05; **p < 0.01; ***p < 0.001.

According to Urbach and Ahlemann (2010), the value of *R*-square should be high enough to get the minimum level of explanatory power. Mental health is influenced by economic crises and perceived job insecurity and presents the value of 0.358 and 0.324 of *R*-square and adjusted *R*-square, respectively (Figure 2). The value of t-statistics must be greater than 1.96 at the 95% confidence level; all the proposed hypotheses followed this threshold value. The obtained figure for f-square is 0.174 which falls at the moderate level of behavior of each construct within the adjusted model. The recommended benchmarks are 0.02, 0.15, and 0.35, considered as small, medium, and large, respectively (Hair et al., 2014). The obtained figure of Q-square is 0.198, which should be greater than 0 from the threshold criterion and measures predictive relevance for a specific construct within the model (Fornell and Larcker, 1981; Chin, 2010; Hair et al., 2013). SRMR showed the value of 0.079 while its NFI value is 0.91 as per a criterion of greater than 0.90.

## Discussion

The available studies are insufficient to assess the secondary impact of COVID-19 in its multiple inflows of waves. Many studies have focused on the consequences of isolation, mental health, shortcomings, and financial instabilities (Douglas et al., 2020; Marsden et al., 2020). As people are facing the threat of job loss or drastic changes at their workplaces, evidence from China has shown that people are directed toward new kinds of addictions such as excessive use of the internet. Statistical measures described that an increase of 46.8% in the usage of internet has been observed while 23% of people have severe internet addictions. Furthermore, people started to abruptly take other injurious materials like alcohol, and alcohol relapse was noted among ex-drinks (Sun et al., 2020).

Despite numerous studies on the combination of fear of economic crisis and perceived job insecurity, this study has proposed a model on recent influential bearings of COVID-19. Firstly, the study described the origin of COVID-19 and showed the pandemic transmission under the consequences of lockdowns and imposed barriers. Due to ongoing pandemics, the study demonstrated that people experienced stress, depression, and anxiety under unusual circumstances. As a result, their attention started to revolve around whether their current positions were safe or not. People often neglect this factor to sustain their current and available opportunities, jobs, and incomes. The conducted study contributes to the body of knowledge on mental instability of working people. Due to the pandemic, these working conditions downgraded people’s mental health and hurt the working environment of the overall workforce (SDG 8).

The study persisted in answering the research questions by highlighting the factors that affected fear of economic crisis and perceived job insecurity, which silently impact the mental and psychological health of an employee (Lind and Van den Bos, 2002), and how emotional labor strategies (surface acting and deep acting) moderate the relationship of fear of economic crisis and perceived job insecurity with mental health (Rehman et al., 2021). The study fulfilled its objective by examining its impact in the context of COVID-19. The purpose of the study is to provide a roadmap about the ongoing pandemic and its resulting fear of economic crisis and perception of job insecurity for senior management to strengthen and revise their policies in the context of emotional labor strategies that can enhance the mental health of organizational members.

This study sustained the results of earlier studies that showed fear of economic crisis can badly damage an employee’s mental health (Frasquilho et al., 2015; Silove et al., 2017; Wang et al., 2021). Similarly, the findings of this study are also in line with the literature about the relationship between perceived job insecurity and mental health (Menéndez-Espina et al., 2019; Zhang et al., 2020). The current study also successfully highlighted the positive impact of emotional labor strategies as per the literature studies (Chi and Grandey, 2019; Rehman et al., 2021). The study’s findings filled the research gap and highlighted the moderating role of emotional labor strategies, which is the main contribution of the study. This moderation impact was never before studied in the literature on COVID-19 and achieving UN SDG 8. The current study has increased the responsibility of policymakers and senior managers’ in designing strategies that constructively improve employees’ behaviors to have a decent work environment. A healthier workforce can be achieved by adopting adequate measures of social protection systems, as employees’ mental health is an asset of each organization, which is the critical determinant of a decent working environment. In this way, organizations should provide training to their concerned managers; managers must provide optimum confidence to their respective employees about the constancy of the existence of their current positions. Upper management should keenly monitor their emotions and expressions to make employees confident enough that their jobs are also secured in the situation of a crisis. Therefore, employees should develop a common practice of putting aside their inner feelings and personal emotions and trying to mitigate the gap between their inner and apparent emotions because their remunerations and continuation of the jobs are highly dependent on the emotional labor strategies.

Professionals like cashiers, call center employees, receptionists, bus drivers, actors, nurses, and teachers, where the employees directly interact with customers and strangers, often need emotional labor strategies for better social interactions. Humans cannot interact with an anonymous audience and put aside their disturbances, inner feelings, and emotions because inner expressions are naturally displayed in facial expressions and body language. Therefore, in uncertain situations, employees are to be confident enough and ready to accept the circumstances as a challenge and try their best to improve their mental health on the way for sustainability. The support and policies at a governmental level can also reduce fears and job insecurities and boost the workforce’s confidence to work effectively. It ultimately creates a decent working environment and leads toward economic growth to achieve the United Nations Sustainable Development Goals.

### Limitations and Future Research Directions

The conducted study has some limitations. Firstly, a small sample and random sampling technique were used due to dependence on social media channels. Second, the imbalanced distribution of gender may cause gender discrimination biases because a significant portion of the respondents was comprised of males (73%). There is the possibility of a change in statistical values with the changing proportion of gender. The limitation of gender discrimination bias can be used as the future direction of the study.

The study narrowed down one aspect of decent work and economic growth of SDG 8 to mental health, job insecurity, and fear of economic crisis. This research opens avenues to research in various aspects of mental health and job insecurities to enhance the well-being of the workplace, such as SDG 8 regarding gender inequality in the workforce discussed by Rai et al. (2019). This could be discussed and analyzed in future research.

Future researchers have an opportunity of evaluating the subject as a whole. Moreover, the current model has the flexibility to be tested and adjusted to analyze variable constructs like moderating impact of motivation.

## Conclusion

COVID-19 was a worldwide crisis that led to the declaration by WHO of a global emergency. The infectious virus is perilous and unique in nature. Several barriers were imposed to control the further dispersion of the virus. People, organizations, institutions, and statutory bodies are suffering various kinds of challenges as a consequence of these barriers. Subsequently, people have undergone various kinds of mental and psychological disorders that have severe impacts. We have concluded the significant negative impact of COVID-19 with the consortium effect of economic crises and job insecurity on mental health. It posits important implications for SDG 8 to consider these variables in order to achieve workers well-being goals by 2030. This study reports the labor strategies as the solution for the adverse effects of COVID-19, economic crises, and job insecurity on mental health. The study proves that deep acting strategies create positive mental health in COVID-19 situations in fear of economic crisis and job insecurities. Surface acting has a minor impact and reduces the severity of independent variables, but deep acting is the significant variable to consider because it affects workers’ mental health and alters the negative impact of adverse circumstances into positive.

### Implications of Study

This study provides solid practical implications for sustainable development goals that are to be achieved by 2030 and theoretical implications by making an addition to the body of existing literature as corporate and service sectors are never studied by the researchers from an emotional labor strategies viewpoint in the perspective COVID-19. SDG 8 deals with decent work and economic growth, and a healthy workforce is imperative to achieve these goals. The study’s results show that governments in underdeveloped countries should develop deep acting strategies to have a mentally strong workforce. In the pandemic, a high rate of job insecurity could be counteracted by deep acting strategy which provide a significantly positive effect on mental health. A mentally strong workforce creates decent work and contributes significantly to economic growth. The policymakers and governmental and international bodies should consider deep acting strategies in workforce policies and to cope with the adverse pandemic and economic situations.

## Data Availability Statement

The original contributions presented in the study are included in the article/supplementary material, further inquiries can be directed to the corresponding author/s.

## Author Contributions

SR contributed to writing, conceptualizing, and analyzing the data. MH performed the literature review and research design. AN contributed to correspondence, discussion, and methodology. AU contributed to defining and writing implications, and overall proofreading. NA performed data collection, analysis, and references. All authors contributed to the article and approved the submitted version.

## Conflict of Interest

The authors declare that the research was conducted in the absence of any commercial or financial relationships that could be construed as a potential conflict of interest.

## Publisher’s Note

All claims expressed in this article are solely those of the authors and do not necessarily represent those of their affiliated organizations, or those of the publisher, the editors and the reviewers. Any product that may be evaluated in this article, or claim that may be made by its manufacturer, is not guaranteed or endorsed by the publisher.
